# Improving Sodium Alginate Films Properties by Phenolic Acid Addition

**DOI:** 10.3390/ma13132895

**Published:** 2020-06-28

**Authors:** Beata Kaczmarek

**Affiliations:** Department of Biomaterials and Cosmetics Chemistry, Faculty of Chemistry, Nicolaus Copernicus University in Toruń, Gagarin 7, 87-100 Toruń, Poland; beata.kaczmarek@umk.pl; Tel.: +48-56-611-4010; Fax: +48-56-654-2944

**Keywords:** sodium alginate, tannic acid, films, food packaging

## Abstract

Currently, packaging materials constitute a group of the most commonly used products. Natural polymers are widely tested as potential packaging materials to replace traditional plastics. Sodium alginate is eco-friendly and reveals effective film-forming properties whereas tannic acid has been proposed as a sodium alginate cross-linker. Thin films of sodium alginate/tannic acid were obtained by solvent evaporation. Interactions between the components were determined as well as the maximum tensile strength and color change after contact with different solutions. Improvement in the physicochemical properties of the obtained films was noticed. Moreover, such films showed antioxidant properties. It may be assumed that materials based on a sodium alginate/tannic acid mixture are promising alternatives to traditional packaging materials.

## 1. Introduction

Currently, global consumption of plastics is higher than 200 million tonnes in the years 1950–2018 (https://www.statista.com). Currently, packaging materials are the most common consumables [1]. They are used to cover food to provide protection, and then disposed of after single use. For this reason, it is important to search for biodegradable materials, which may be potentially used to effectively protect food but also are eco-friendly [2,3,4].

Numerous potentially applicable materials have already been tested. They are obtained mainly from polymers, both natural and synthetic ones, because they can be easily manufactured [5]. Various synthetic polymers, e.g., polyethylene terephthalate (PET), polyvinylchloride (PVC), polyethylene (PE), polypropylene (PP), polystyrene (PS) or polyamide (PA) are currently used [6,7,8]. They are characterized by features required for packaging materials; however, they are non-biodegradable.

Currently, a trend called “from nature to nature” is observed in the field of materials science Natural polymers are eco-friendly and biodegradable [9,10,11,12]; nevertheless, they have poor physicochemical properties [13]. Thereby, they may be considered as raw materials for packaging production after initial modifications, which lead to improving their properties [14,15,16].

Sodium alginate is a polysaccharide, which has found a wide range of applications to obtain usable materials [17]. The main disadvantage of this biopolymer is its low stability under wet conditions and high sensitivity to degradation processes. Various modifications of sodium alginate have been considered, e.g., adding metal ions such as (Cd(II), Cu(II) and Pb(II)) [18] or combining with polymers, i.e., cellulose [19] or chitosan [20]. Phenolic acid addition is a novel method to improve properties of films obtained from sodium alginate.

Phenolic acids are naturally derived compounds that show useful properties such as antimicrobial [21], antiviral [22] and even anticancer activity [23]. Moreover, they are also good cross-linkers for proteins and/or polysaccharides [24,25,26]. Tannic acid is one of phenolic acids and it may be easily obtained from plants [27]. It has been previously considered as an effective cross-linker for chitosan-based mixtures [28,29,30]. In the present study, we would like to discuss improving the properties of sodium alginate films by adding tannic acid as a cross-linker. It can be stated that the presence of tannic acid enhances the physicochemical properties of materials based on sodium alginate and also provides novel properties such as antioxidant activity.

## 2. Materials and Methods

### 2.1. Samples Preparation

Sodium alginate (SA) and tannic acid (TA) were purchased from Sigma-Aldrich (Darmstadt, Germany). They were both dissolved in 0.1 M acetic acid. The film preparation method was also studied for water solutions; however, such films were not stable under wet conditions.

Sodium alginate solution was mixed with tannic acid in the weight ratio of 90/10, 80/20 and 70/30. A lower sodium alginate content did not cause the film formation. SA and TA were mixed for 2 h with a magnetic stirrer. Then, they were placed on the plastic holders and left until the solvent evaporated. After 48 h, thin solid films were obtained. For all the samples, the thickness measured with a gauge (Sylvac, Valbirse, Switzerland) was 0.021 ± 0.007 mm.

### 2.2. Infrared Spectroscopy (FTIR-ATR)

FTIR-ATR spectra were measured for each type of film in the range of 1800–800 cm^−1^ at the resolution of 4 cm^−1^ with the spectrometer (Nicolet iS110, Shimadzu, Kyoto, Japan) equipped with a diamond crystal. The spectra were taken with 64 scans.

### 2.3. Mechanical Parameters

Maximum tensile strength, Young’s modulus and elongation at the break (*n* = 5) were determined with the use of a testing device Z.05 (Zwick/Roell, Ulm, Germany) with the initial force 0.1 N and the velocity of 5 mm/min. The measurement was carried out under room conditions. 

### 2.4. Wettability

The surface wettability was measured by the glycerin droplet placement on the film. The measurement was made with a goniometer equipped with a system of drop shape analysis (DSA 10 Control Unit, Krüss, Hamburg, Germany). The contact angle was measured immediately after the droplet placement, and also after 5, 10 and 15 s. Changes in the contact angle over time were observed. 

### 2.5. DPPH Radical Scavenging Assay

Antioxidant properties of films were determined using the DPPH reagent (2,2-Diphenyl-1-picrylhydrazyl, free radical, 95%; Alfa Aesar, Karlsruhe, Germany) [31]. A 250 µM solution of DPPH in methyl alcohol as a solvent was prepared for the reaction. Samples (1 cm × 1 cm) of each film were placed in a 12-well plate and filled with 2 mL of a DPPH solution. They were left without exposure to light for 1 h. The tested samples were replicated five times. The control sample was a DPPH solution left on the plate without contact with the films. After incubation, a spectrophotometric measurement was made at 517 nm (UV-1800, Shimadzu, Reinach, Switzerland). The antioxidant activity was calculated from the formula:

(1) $$RSA\%=\frac{Abs_{DPPH}-Abs_{PB}}{Abs_{DPPH}}\times 100$$

where: *Abs_DPPH_* is the absorbance of the DPPH solution without contact with the materials and *Abs_PB_* is the absorbance of the DPPH solution after contact with the material being tested

### 2.6. Color Change 

Each film sample was cut into 1 cm × 1 cm squares. These were placed in a 12-well plastic holder. The droplets of three liquids were placed on their surface: 2 mol/L HCl, 2 mol/L NaOH and water. Color variations were measured after 1 h using a colorimeter (Corneometer CL 400, Courage, Khazaka, Köln, Germany). The color parameter L (which describes the sample lightness), a (along the X axis red (+) to green (−)) and b (along the Y axis yellow (+) to blue (−)) were evaluated (Sun, Jiang, Wu, Tong and Pang, 2020;). The total color difference (ΔE) was then calculated using the equation:

(2) $$\Delta E={\left(\Delta L^{2}+\Delta a^{2}+\Delta b^{2}\right)}^{0.5}$$

where: *ΔL = L − L_0_, Δa = a − a_0_, Δb = b − b_0_* and *L_0_, a_0_* and *b_0_* are color values of the control

### 2.7. Water Vapor Permeation Rate (WVPR)

The water vapor permeation rate (WVPR) of each film type was investigated. A determined amount of dried calcium chloride as a desiccant was placed in the plastic container of 25 mm diameter. The top of the container was covered with the tested films (*n* = 3). The containers with calcium chloride without covers were left as control samples. After 3 days, the films were removed and the WVPR was calculated using the equation:

(3) $$WVPR\;\left[g\cdot \;m^{2}\cdot \;h^{-1}\right]=\frac{W}{A\;\cdot t}\times 100\%$$

where: *W*—the gain weight (g); *A*—area of the film cover and *t*—time (*h*)

### 2.8. Oxygen Permeability

Oxygen permeability of the polymer films was evaluated by measuring the amount of dissolved oxygen in the distilled water [32]. Deionized water was boiled for 10 min to remove the whole dissolved oxygen. Plastic containers of 4 cm diameter were filled with 50 mL of oxygen free deionized distilled water and covered with the tested films. Containers without covers were left as control samples. Samples were stored under room conditions for 24 h. Each kind was evaluated in triplicate. The amount of dissolved oxygen was calculated by the Winkler’s titration method.

## 3. Results

### 3.1. Infrared Spectroscopy (FTIR-ATR)

**Figure 1:**
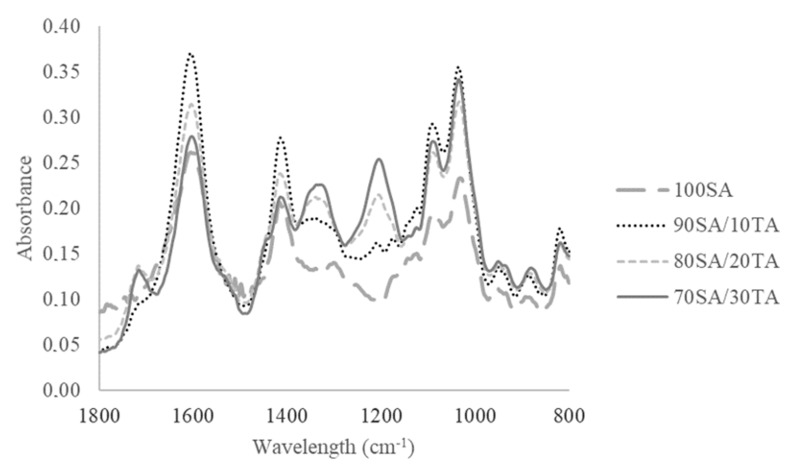
FTIR-ATR spectra of thin films.

FTIR-IR spectra provide data on the interactions between sodium alginate and tannic acid on the molecular level (Figure 1). According to the performed research results, tannic acid addition does not influence the shape of the peaks. The characteristic peaks obtained from sodium alginate are noticed on the spectra of hydroxyl and carboxylic functional groups. Asymmetric and symmetric stretching vibrations of the carboxylate salt ions are observed in 1598 and 1407 cm^−1^ [31]. Peaks in 1035 and 1087 cm^−1^ were attributed to the C–O stretching vibration of the pyranose ring and the C–O stretching with contributions from C–C–H and C–O–H deformation [33]. New peaks were observed on the spectra of films with tannic acid when compared to the spectrum of pure sodium alginate in 1706 cm^−1^ from C=O groups. The peak from the O–H group in 1197 and 1320 cm^−1^ decreased after the tannic acid addition as a result of hydrogen bonds formation between the film components [34].

### 3.2. Mechanical Parameters

**Figure 2:**
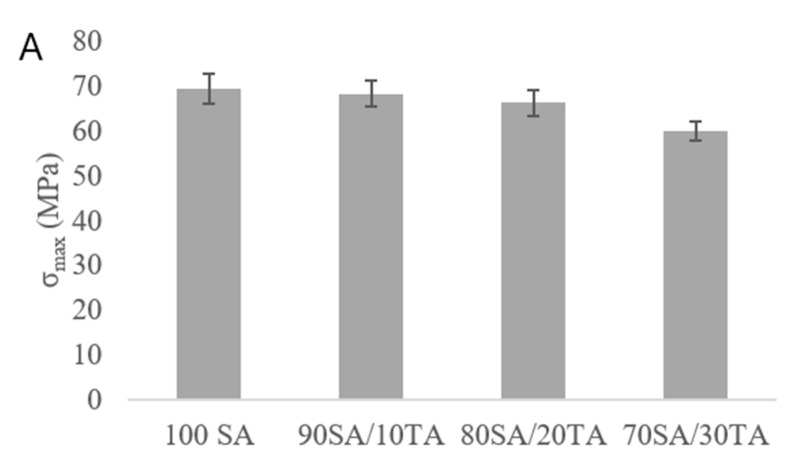

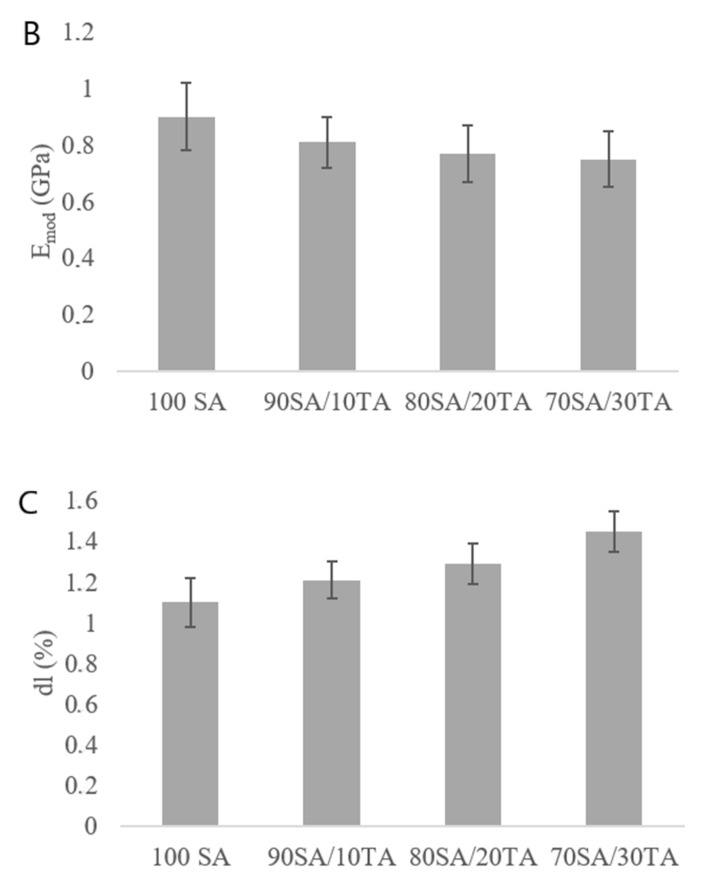
Mechanical parameters of sodium alginate/tannic acid films. (**A**) Maximum tensile strength, (**B**) Young’s modulus and (**C**) elongation at break.

Mechanical parameters such as maximum tensile strength, Young’s modulus and elongation at the break were considered for the films proposed as packaging materials (Figure 2). The addition of tannic acid had no significant influence on the maximum tensile strength. A slight decrease in the maximum tensile strength was observed after the complex formation when compared to a pure sodium alginate-based film. For all the tested samples, the mechanical parameter was in the range around 70 MPa. Young’s modulus, the value that determines a material stiffness, decreased with an increasing amount of tannic acid. As the decrease in E_mod_ was observed, it suggests that the films were more flexible after tannic acid addition than without it. At the same time, the elongation at the break increased, which confirms the increase in flexibility after tannic acid addition to sodium alginate. 

### 3.3. Wettability

Wettability of the film surface is a parameter useful when examining the material behavior in contact with wet food. The contact angle for glycerin as the hydrophilic solution was measured immediately after the droplet placement and after 5, 10 and 15 s (Table 1). The change in the film hydrophilicity over time was observed for all the films. However, a pure sodium alginate film showed the difference in the contact angle of around 23 degrees in 15 s as a result of the solution soaking. It suggests that the film was sensitive to the hydrophilic conditions. However, tannic acid addition reduced the change and for 70SA/30TA the difference was 11 degrees. It suggests that the surface was more resistant to the hydrophilic conditions. 

### 3.4. DPPH Radical Scavenging Assay

Antioxidant properties of novel packaging materials have attracted special attention since they are promising alternatives to traditional ones. Tannic acid has been proposed as a cross-linker but it also possesses antioxidant properties. The film with 30% tannic acid addition showed three times higher antioxidant activity than the pure sodium alginate film (Table 2). 

### 3.5. Color Change

**Figure 3:**
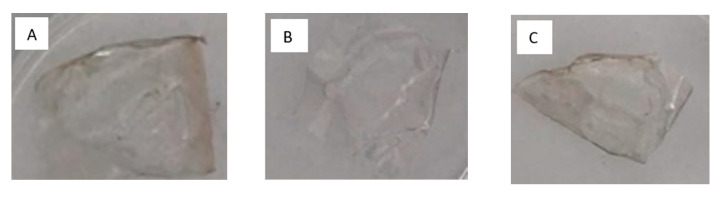

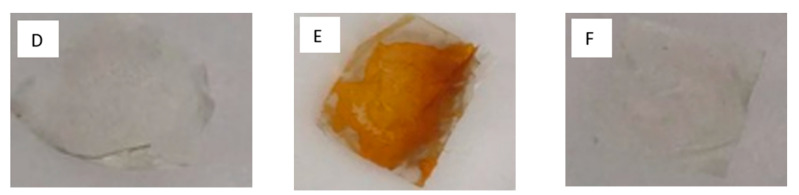
Images of films based on sodium alginate in (**A**) HCl, (**B**) NaOH and (**C**) water and 70SA/30TA in (**D**) HCl, (**E**) NaOH and (**F**) water.

The film color change was not visually observed. Tannic acid addition to sodium alginate resulted in increasing opacity together with an increase in the amount of polyphenol acid after contact with HCl solution (Table 3). NaOH placement on the film surface caused a significant color change (Figure 3), which was confirmed by colorimetric measurement. It suggests that the proposed film should not be used for food characterized by alkaline pH. The color of films contacted with water did not change.

### 3.6. Water Vapor Permeation Rate (WVPR)

The results of WVPR are listed in Table 4. The water vapor permeation rate is an important factor for the materials applied as dressings because the moist environment of a wound should be maintained to support the healing processes [35]. For the control sample (a container without the film), the WVPR was 82.70 g/m^2^/h. For the films based on unmodified sodium alginate, the WVPR was 16.13 g/m^2^/h and with tannic acid addition the WVPR was in the range of 21.45–24.29 g/m^2^/h. An increasing amount of tannic acid raised the water vapor permeation rate.

### 3.7. Oxygen Permeability

**Figure 4:**
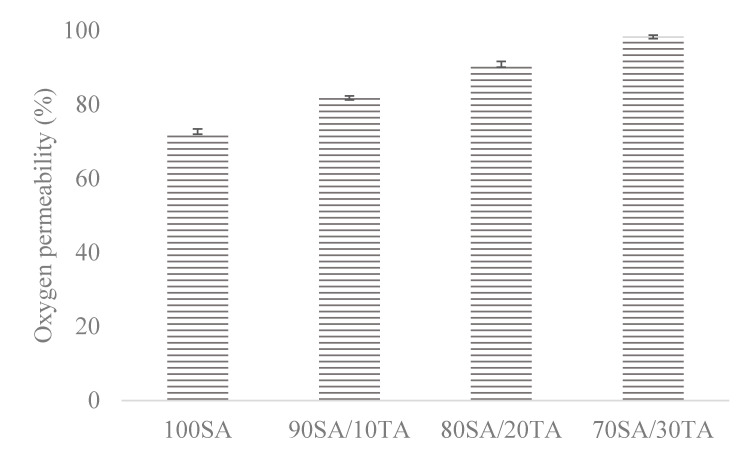
Oxygen permeability through films based on sodium alginate modified by tannic acid addition. Results are presented as percentage permeability vs. control (permeability 100%).

Oxygen permeability was determined for each type of sodium alginate/tannic acid film (Figure 4). An increased tannic acid concentration results in an increase in oxygen permeability proportionally to TA content. Oxygen permeability 100% was customized for the control sample (with no covering). The films based on 70SA/30TA showed an oxygen permeability value at almost 100%.

## 4. Discussion

### 4.1. Infrared Spectroscopy (FTIR-ATR)

FTIR-IR spectra proved the presence of sodium alginate functional groups, i.e., hydroxyl and carboxylic ones. After the addition of tannic acid, the new peak was observed from the C=O group. The obtained spectra suggest that hydrogen interactions take place between sodium alginate and tannic acid any covalent bonding and novel complexes of SA/TA are formed via strong hydrogen interactions.

### 4.2. Mechanical Parameters

Any modifications of biopolymeric-based materials should not lower important parameters such as the mechanical ones. It may be assumed that tannic acid interacts with sodium alginate; however, weak hydrogen interactions slightly lower the maximum tensile strength and, for all the tested samples, the value was around 70 MPa. The loss in tensile strength is attributed to the change of the polymeric network caused by the incorporation of tannic acid. Common traditional plastics present the tensile strength ca. 10 MPa for LDPE (low density polyethylene) up to 70 MPa for PET [36]. On the other hand, biodegradable chitosan-based films proposed for packaging purposes fabricated by Jakubowska et al. [37] showed the tensile strength value dependence on the deacetylation degree (DD). Chitosan with DD = 72% showed the tensile strength ca. 66.1 MPa and for chitosan with DD = 83% ca. 107 MPa. Sodium alginate films enriched with tannic acid may be an eco-friendly alternative in the future for products made of PET. Lower stiffness is necessary when a material is used as packaging to cover food and which should not have any crashes on its surface. The comparison of the Young’s modulus value for sodium alginate/tannic acid films with those for other sodium alginate-based films provides evidence that the natural compounds addition leads to obtaining materials of stiffness lower than that of, e.g., graphene oxide [38]. Sodium alginate/tannic acid films showed a lower Young’s modulus value than that for chitosan-based materials [37] as they were more flexible.

### 4.3. Wettability

The value of the contact angle is an important criterion for predicting possible interactions of a packaging material with food. Tannic acid addition results in the decrease in the polar liquid soaking rate when compared to that for pure sodium alginate-based films as it interacts with hydrophilic groups of the polymer. The contact angle for the polar liquid on the PET surface was determined by Gotoh et al. [39]. The initial angle for glycerin was around 83° and, after a short period of time, it decreased to 52°. Yanghuang et al. [40] reported the contact angle for water on the PET surface equal to 72°. The differences were related to the type of liquid used for the study. Similar observations were made in the correlation for sodium alginate/tannic acid-based films where it was equal to 90°, 85° and 76° for 10%, 20% and 30% tannic acid content, respectively.

### 4.4. DPPH Radical Scavenging Assay

Novel packaging materials should present antioxidant properties to slow down the food auto-oxidation process. On the one hand, tannic acid is added to cross-link sodium alginate but also to function as an active compound on the other. It provides the antioxidant properties to the films finally produced from the sodium alginate/tannic acid complexes. The antioxidant activity increased with the increasing content of tannic acid. PET does not show the antioxidant activity but has to be modified by, e.g., the coating formation from rosemary and clove extracts [41]. Dou et al. [42] modified adding sodium alginate gelatin and tea polyphenols. An increase in antioxidant activity was observed with an increasing content of tea polyphenols. Without tea polyphenols, the RSA% was around 5% and rapidly increased to around 37% after active compounds addition. RSA% obtained for sodium alginate/tannic acid-based films were ca. 5.87% for films without TA and 28.42%, 43.74% and 50.50% for 10%, 20% and 30% addition, respectively. It provides the significant advantage in sodium alginate/tannic acid-based film application in the food industry. 

### 4.5. Color Change

Color change of packaging materials is an important aspect when customers’ preferences are concerned [43]. The parameter L (lightness) for the control sample was higher than that for he tested film after contact with media. It suggests that the film darkens as a result of getting in contact with liquids (negative values). However, the change is not visible with the naked eye (Figure 3). The ISO 11664-4:2008 standard states that *ΔE* > 5 is noticed as a color change [44]. The lowest visible change was thereby noticed for films with 30% tannic acid content for the acid solution. *ΔE* > 5 was also noticed when water was applied on the material surface, however, the presence of tannic acid did not influence the color as a sodium alginate/tannic acid complex was formed. The proposed materials cannot be applied for basic solutions since in such cases the color change is significant. To improve resistance to color change, films may be coated with a protective layer. It will be further investigated.

Based on the obtained results, it may be assumed that the presence of tannic acid enhances the physicochemical properties of materials based on sodium alginate and provides novel features such as antioxidant activity.

### 4.6. Water Vapor Permeation Rate (WVPR)

The water vapor permeability rate is a crucial parameter when considering a material as a potential packaging one. The water permeability process improves food quality. There are parameters regarded as the model ones. The modification of sodium alginate by tannic acid addition results in the WVPR increase as tannic acid bears many hydroxylic groups, which are able to bind water. As a result, the improvement in the WVPR in comparison to the rate for films without TA was noticed. The WVPR values for commercial materials were 0.15 g/m^2^/h for PET and 0.33 g/m^2^/h for LDPE. The WVPR values for SA/TA films were much higher than for commercial plastics; thereby, they may not be used for dry food storage.

### 4.7. Oxygen Permeability

Oxygen presence may cause oxidation of the stored food and, as a result, change its color, odor, nutritional value, content, etc. Packaging materials presenting low oxygen permeability are useful to improve food quality. As a hydrophilic polymer, sodium alginate turned out to be a good oxygen barrier, which corresponded with the results obtained by Sothornvit et al. [45]. The addition of tannic acid as a cross-linker resulted in blocking hydrophilic groups, and so, the oxygen permeability rate increased. For such a reason, lower tannic acid concentration resulted in the preparation of a material showing better oxygen barrier properties.

## 5. Conclusion

Sodium alginate presents film-forming properties. However, materials based on a pure polysaccharide are not stable under wet conditions. Tannic acid was successfully added to sodium alginate as a cross-linker owing to hydrogen interactions present. Films with tannic acid showed antioxidant properties and they may be alternatives for traditional packaging materials. The proposed sodium alginate/tannic based materials can be considered as materials applicable for food storage; nevertheless they are useful for products characterized by acidic or neutral pH. It may be assumed that tannic acid-enriched films based on sodium alginate are promising food packaging materials; however, they need further modifications to improve the inhibition of oxygen permeability.

## Figures and Tables

**Table 1:** Glycerin contact angle on the films surface.

Specimen	0 s	5 s	10 s	15 s
100SA	73.67 ± 1.16	67.33 ± 0.47	60.73 ± 1.66	51.17 ± 1.25
90SA/10TA	90.10 ± 1.55	78.85 ± 2.05	74.70 ± 0.42	66.90 ± 0.28
80SA/20TA	85.00 ± 1.47	76.97 ± 1.55	73.97 ± 1.58	68.20 ± 0.94
70SA/30TA	76.30 ± 1.40	70.15 ± 0.89	68.80 ± 1.77	65.90 ± 0.72

**Table 2:** Antioxidant activity (RSA%) of sodium alginate/tannic acid films.

Specimen	RSA%
100SA	5.87 ± 1.12
90SA/10TA	28.42 ± 0.98
80SA/20TA	43.74 ± 1.07
70SA/30TA	50.05 ± 0.94

**Table 3:** Color parameters (*L—lightness, a—red (+) to green (−), b—yellow (+) to blue (−b)* and *ΔE—total color difference*) of the films.

Specimen	Color Parameter	Solution		
		HCl	NaOH	Water
100SA	*L*	−7.73 ± 1.24	−5.90 ± 1.03	−7.29 ± 0.91
	*a*	2.15 ± 0.78	2.46 ± 0.77	2.22 ± 0.73
	*b*	6.54 ± 1.09	1.69 ± 0.82	7.08 ± 1.02
	*ΔE*	10.35 ± 0.91	6.61 ± 0.67	10.40 ± 0.88
90SA/10TA	*L*	−6.98 ± 0.68	−13.05 ± 1.56	−10.27 ± 0.62
	*a*	2.48 ± 0.90	7.90 ± 1.71	3.17 ± 1.08
	*b*	5.00 ± 0.65	30.98 ± 0.76	2.00 ± 0.77
	*ΔE*	8.94 ± 0.61	34.53 ± 0.61	10.93 ± 1.04
80SA/20TA	*L*	−8.25 ± 0.44	−12.11 ± 1.11	−7.10 ± 0.66
	*a*	3.46 ± 1.31	12.50 ± 0.95	3.22 ± 1.01
	*b*	3.91 ± 0.98	31.89 ± 0.74	6.42 ± 0.99
	*ΔE*	8.76 ± 1.01	36.32 ± 1.03	10.09 ± 0.73
70SA/30TA	*L*	−7.02 ± 0.94	−18.39 ± 0.98	−6.48 ± 0.96
	*a*	1.70 ± 0.67	14.79 ± 1.03	2.58 ± 1.07
	*b*	3.23 ± 0.54	24.88 ± 0.77	4.86 ± 0.95
	*ΔE*	7.91 ± 1.09	34.29 ± 0.54	10.05 ± 1.43

All data are shown as mean ± standard deviation (SD).

**Table 4:** Water permeability (WVPR) of films based on sodium alginate and tannic acid.

Specimen	WVPR (g/m^2^/h)
100SA	16.13
90SA/10TA	21.45
80SA/20TA	22.54
70SA/30TA	24.29
